# Optimal Multiphase Computed Tomographic Angiography-based Infarct Core Estimations for Acute Ischemic Stroke

**DOI:** 10.1038/s41598-019-51708-6

**Published:** 2019-10-23

**Authors:** Seong-Joon Lee, Woo Sang Jung, Mun Hee Choi, Ji Man Hong, Jin Soo Lee, Jin Wook Choi

**Affiliations:** 1 Department of Neurology, Ajou University School of Medicine, Ajou University Medical Center, Suwon, South Korea; 2 Department of Radiology, Ajou University School of Medicine, Ajou University Medical Center, Suwon, South Korea

**Keywords:** Outcomes research, Stroke

## Abstract

We evaluated the best methods for predicting various infarct core thresholds for endovascular treatment of ischemic stroke using parameters obtained by multiphase computed tomographic angiography (mCTA). Consecutive patients evaluated for endovascular treatment who concomitantly underwent mCTA and stroke magnetic resonance imaging (MRI) were analyzed. The ability of CTA-based collaterals (single-phase [sCTAc] and multiphase [mCTAc]) and ASPECTS or their combined interpretation for the selection of patients with cores of <31 ml and <70 ml, and ≥100 ml, were compared. In the total 142 patients, the combined interpretation of collateral scores and ASPECTS score indicated significant added benefit for the prediction of smaller infarct volume thresholds (<31 ml) compared to ASPECTS alone. Selection of cases that satisfied both sCTAc 3–5 and ASPECTS 6–10 had the optimal predictive capability and inter-rater reliability. While the combined interpretation did not provide a significant added benefit for the prediction of larger infarct volume thresholds, sCTAc 0–2 and mCTAc 0–2 performed as well as ASPECTS 0–5 in prediction of core volumes ≥100 ml with better inter-rater reliability. sCTA and mCTA can improve the selection of patients for EVT by more accurately predicting lower infarct core volume cutoffs. When excluding patients with large infarct cores, they can improve inter-rater reliability.

## Introduction

Pretreatment ischemic core volume is an independent predictor of functional outcomes in acute ischemic stroke with proximal arterial occlusion^1^. While the concept of mismatch profiles was used in some of the endovascular treatment (EVT) trials^2,3^, the extended time window trials emphasized the importance of lower infarct cores with clinical core mismatch instead^4^. The concept of late window paradox further helped reveal that ischemic core volume can also represent progression speed^5^. Accordingly, infarct core volume is one of the most important variables for determining the outcome of EVT. While extended time window trials outlines lower infarct volumes important for identification^6,7^, the upper limit of infarct cores that still maintains the treatment effect of EVT has yet to be determined, and some studies have suggested significant treatment effects even in larger infarct volumes up to 133 ml^8^. An ideal imaging protocol should address both the lower and higher infarct volume thresholds.

While the infarct volume may be most easily determined using magnetic resonance imaging (MRI) diffusion weighted images (DWI), recent endovascular trials have rarely used DWI volume to determine patient eligibility because of the strong emphasis on limiting delays^9^. Computed tomographic (CT) perfusion has been increasingly used instead^2,3^, but perfusion imaging and interpretation may not be available in a substantial number of institutions.

CT angiography (CTA)-based collateral imaging is another useful modality, and recent studies have reported the value of multiphase CTA (mCTA)-based collateral grading (mCTAc)^10^. Similar to CT perfusion, mCTA can also be used to predict tissue fate regionally^11^ through visual grading. The Alberta stroke program early CT score (ASPECTS) is another parameter obtained from initial CTA, and it is an excellent clinical tool for core evaluation that can be applied easily and has good predictive power for treatment outcomes^12^.

For mCTA to be used as the sole modality for patient selection for EVT, its predictive ability should be sufficient for both smaller and larger infarct volume thresholds. Accordingly, in acute ischemic stroke patients with large vessel occlusion, we evaluated whether parameters obtained in mCTA can appropriately predict both smaller and larger infarct volume thresholds.

## Methods

### Study population

Patients treated between February 2016 and June 2018 from a prospectively designed institutional registry were retrospectively included in this study. Consecutive patients evaluated for reperfusion treatment due to acute stroke were screened. From this database, patients were included in the analysis if they fulfilled the following eligibility criteria: (1) consecutive imaging with mCTA protocol and stroke MRI; (2) documented large vessel occlusion of intracranial anterior circulation (internal carotid artery, middle cerebral artery M1, and M2); (3) availability of 3-month functional outcomes. The data collection protocol was approved by the Ajou University hospital Institutional Review Board (AJIRB-MED-MDB-19-273) and implemented in accordance with the ethical standards of the 1964 Declaration of Helsinki and its later amendments. The need for written informed consent was waived by the Ajou University hospital Institutional Review Board given the retrospective nature of the study.

### Reperfusion protocol

Intravenous thrombolysis was given to patients that presented within 4.5 h of onset and when indicated. EVT was considered in all patients with an expected onset to puncture time <8 h if a large artery occlusion was observed on CTA with corresponding stroke signs. Patients with large core volumes were excluded according to ASPECTS scores (usually <4). For patients that presented after the 8 h limit, EVT was performed on a case-by-case basis depending on potentially salvageable tissue identified on non-enhanced CT (NECT) and stroke MRI. The CT angiography-based collateral imaging was not actively used for patient selection during this period. The type of EVT procedure was chosen at the discretion of the treating physician, and modern thrombectomy procedure was performed for all patients.

### Imaging analysis

Detailed imaging protocols for multiphase CTA and stroke MRI are included in the Supplementary Methods. A baseline ASPECTS score was measured on NECT. The single-phase CTA collaterals (sCTAc) were graded from 0–5 by analysis of the first phase of the mCTA images, with comparisons made between the ischemic and asymptomatic hemispheres. The sCTAc grades were categorized as follows: Grade 5, increased or normal prominence and extent of pial vessels; Grade 4, slightly reduced prominence and extent of pial vessels; Grade 3, moderately reduced prominence and extent of pial vessels; Grade 2, decreased prominence and extent and regions with no vessels, Grade 1, just a few vessels visible; Grade 0, no vessels visible. The mCTAc was graded from 0–5 by analysis of the obtained three-phase images, also with a comparison between the ischemic and asymptomatic hemispheres. The mCTAc grades were categorized as follows: Grade 5, no delay and normal or increased prominence of pial vessels/normal extent; Grade 4, delay of one phase in the filling of peripheral vessels, but prominence and extent is the same; Grade 3, delay of two phases in the filling of peripheral vessels or a one-phase delay and a significantly reduced number of vessels; Grade 2, delay of two phases in the filling of peripheral vessels and decreased prominence and extent, or a one-phase delay and some ischemic regions with no vessels; Grade 1, just a few vessels visible in any phase; Grade 0, no vessels visible in any phase)^10^. For the first 74 cases, grading was performed by two independent readers (SJL, WSC) who were blinded to clinical data using an institutional picture archiving and communication system. The different imaging modalities were reviewed on different dates to minimize bias. Discordance of multimodal scoring between the two readers was resolved by consensus. Based on this consensus, the readings of the remaining cases were performed by a single reader (SJL). The inter-rater reliability (IRR) of specific cutoff points of mCTA-obtained parameters were further evaluated using kappa values.

The MRI-based infarct core volume was calculated quantitatively with a threshold ADC value of 600 × 10^−6^ using commercial automatic software (NordicICE; NordicNeuroLab, Bergen, Norway) (JWC). A small core volume of <31 ml^6^ and intermediate core volume of <70 ml^3^ was used for the selection of patients for EVT, and a large core threshold of ≥100 ml^13^ was used for the exclusion of patients not likely to benefit from mechanical thrombectomy.

### Statistical analysis

A receiver operating characteristic (ROC) curve analysis was performed to compare individual and combined imaging modalities with regard to predicting volume thresholds. DeLong’s method was used to compare the area under the curve (AUC) obtained from the ROC curve analysis. Based on the ROC curve analysis, the best cutoff points of individual and combined parameters for the prediction of volume thresholds and predictive power were evaluated using Youden’s index. The IRR of specific cutoff points of mCTA-obtained parameters were further evaluated. In all analyses, *P* < 0.05 was taken to indicate statistical significance. Statistical analyses were performed using SPSS software (version 23; IBM, Chicago, IL).

### Ethics statement

The data collection protocol was approved by the Ajou University hospital Institutional Review Board (AJIRB-MED-MDB-19-273) and implemented in accordance with the ethical standards of the 1964 Declaration of Helsinki and its later amendments. The need for written informed consent was waived by the Ajou University hospital Institutional Review Board.

## Results

### Clinical characteristics

**Figure 1:**
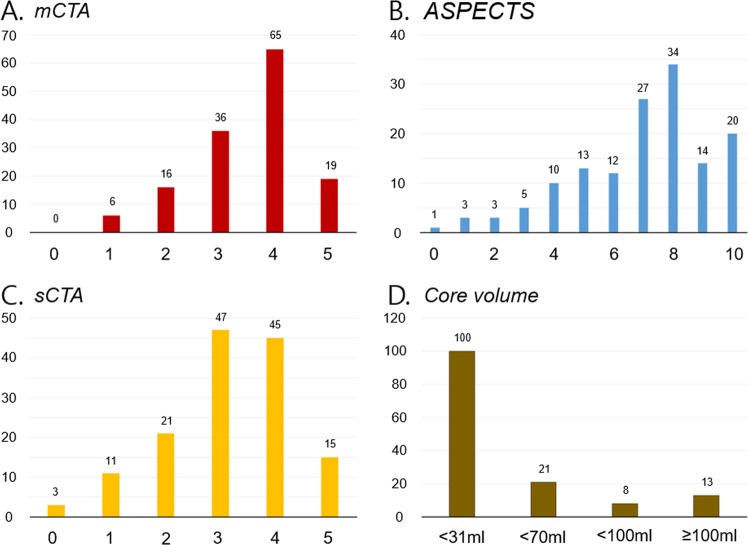
Distribution of mCTA collateral scores, ASPECTS scores, and MR ADC volume. mCTA, multiphase computed tomographic angiography collateral score; ASPECTS, Alberta stroke program early CT score; sCTA, single-phase CT angiography collateral score.

In total, 142 patients were included in the analysis. Among them, intravenous thrombolysis was performed in 62 (43.7%), and EVT was performed in 115 (81.0%). The mean time from onset to CT was 249 ± 239 min and the mean time from CT to MRI was 51.6 ± 12.3 min. Good outcomes were achieved in 75 cases (52.8%) (Supplementary Table 1**)**. The factors associated with functional outcomes in the EVT subgroup are outlined in Supplementary Tables 2 and 3. The distribution of ADC infarct core volumes, ASPECTS scores, sCTAc grades, and mCTAc grades of the patients included in the study are shown in Fig. 1. Because patients were evaluated in the acute phase of presentation, a large number of patients presented with infarct cores <31 ml (100/142, 70.4%), while a small number of patients (13/142, 10.6%) presented with ADC volumes ≥100 ml. The median value for ADC-measured infarct core was 13.65 ml (interquartile range [IQR]: 7.585–42.6175), 7 [IQR: 5.75–8.0] for ASPECTS, 3 [IQR: 2.75–4.0] for sCTAc grading, and 4 [IQR: 3.0–4.0] for mCTAc grading. The validity of the parameters for predicting 3-month good outcomes based on the AUC of the ROC curves was 0.636 (0.544–0.728) for ASPECTS, 0.583 (0.486–0.680) for sCTAc, 0.614 (0.521–0.707) for mCTAc, and 0.636 (0.544–0.727) for ADC volumes.

### ROC curve analysis to determine the statistical significance of combining ASPECTS and collateral scores

Table 1 presents the results of the ROC curve analysis of parameters obtained by mCTA for predicting infarct core thresholds. For the prediction of low infarct volumes, ASPECTS combined with both sCTAc and mCTAc grades had AUC values of 0.898 (*P* = 0.002) and 0.897 (*P* = 0.003), respectively, which were significantly higher than that of ASPECTS alone (0.848). There was no significant added benefit in combining ASPECTS and collateral grades for intermediate and high infarct volumes.

**Table 1:** Predictive power of imaging parameters in predicting various infarct core thresholds.

	Core < 31 ml		
	AUC	95% CI	*P*
ASPECTS	0.848	0.778–0.919	Ref.
sCTAc	0.828	0.748–0.909	0.573
mCTAc	0.809	0.722–0.896	0.308
ASPECTS + sCTAc	0.898	0.839–0.955	0.002
ASPECTS + mCTAc	0.897	0.839–0.955	0.003
	**Core** < **70** **ml**		
ASPECTS	0.875	0.787–0.963	Ref.
sCTAc	0.857	0.785–0.930	0.637
mCTAc	0.864	0.774–0.953	0.787
ASPECTS & sCTAc	0.913	0.853–0.981	0.109
ASPECTS & mCTAc	0.918	0.853–0.972	0.09
	**Core** ≥ **100** **ml**		
(10 – ASPECTS)	0.926	0.836–1.000	Ref.
(5 – sCTAc)	0.925	0.864–0.986	0.979
(5 – mCTAc)	0.959	0.920–0.998	0.392
(10 – ASPECTS) & (5 – sCTAc)	0.956	0.911–1.000	0.340
(10 – ASPECTS) & (5 – mCTAc)	0.976	0.952–1.000	0.189

Combination of ASPECTS scores and collateral scores achieved higher predictive capability for low infarct volumes <31 ml, as shown by significantly higher AUC values.

ASPECTS, Alberta stroke program early CT score; AUC, area under the curve; ADC, apparent diffusion coefficient; sCTAc, single-phase CT angiography collateral; mCTAc, multiphase CT angiography collateral.

### Predictive performance of mCTA parameters and IRR

Appropriate cutoff values were generated using ASPECTS, sCTAc grades, and mCTAc grades (Table 2). For the lower infarct volume threshold (<31 ml), a combination of sCTAc grades 3–5 and ASPECTS scores 6–10 yielded the highest sensitivity (86.0%) and specificity (83.3%) with reasonable IRR (κ = 0.758), followed by a combination of mCTAc grades 3–5 and ASPECTS scores 6–10 (91.0%, 73.8%, and 0.773, respectively).

**Table 2:** Predictive performance of mCTA collaterals, sCTA collaterals, ASPECTS, and combination of collateral imaging and ASPECTS in predicting core volume thresholds of <31 ml, <70 ml, and ≥100 ml, and its inter-rater reliability.

	Sensitivity	specificity	PPV	NPV	Y-index	Kappa
**Core < 31 ml**						
ASPECTS 8–10	60.0	81	88.2	46	141	0.384
ASPECTS 6–10	92.0	64.3	86	77.1	156.3	0.440
mCTAc 4–5	74.0	76.2	88.1	55.2	150.2	0.466
mCTAc 3–5	98.0	47.6	81.7	90.9	145.6	0.787
sCTAc 4–5	56.0	91.5	93.3	46.3	147.5	0.635
sCTAc 3–5	91.0	61.9	85.1	74.3	152.9	0.796
mCTAc 4–5 & ASPECTS 6–10	69.0	88.1	93.2	54.4	157.1	0.595
mCTAc 3–5 & ASPECTS 6–10	91.0	73.8	89.2	77.6	164.8	0.773
sCTAc 4–5 & ASPECTS 6–10	54.0	90.5	93.1	45.2	144.5	0.661
sCTAc 3–5 & ASPECTS 6–10	86.0	83.3	92.5	85.2	169.3	0.758
**Core < 70 ml**						
ASPECTS 6–10	85.8	81.8	96.3	51.4	167.6	0.440
ASPECTS 4–10	99.2	50	91.5	91.7	149.2	0.420
mCTAc 4–5	67.5	86.4	96.4	32.8	153.9	0.466
mCTAc 3–5	93.3	63.6	93.3	63.6	156.9	0.787
mCTAc 2–5	100	27.3	88.2	100	127.3	0.648
sCTAc 4–5	50	100	100	26.8	150	0.635
sCTAc 3–5	83.3	68.2	93.5	42.9	151.5	0.796
sCTAc 2–5	95.8	40.9	89.8	64.3	136.7	0.426
**Core ≥ 100 ml**						
ASPECTS 0–5	92.3	82.2	34.3	99.1	174.5	0.440
ASPECTS 0–3	69.2	97.7	75	96.9	166.9	0.420
ASPECTS 0–1	30.8	100	100	93.5	130.8	-
mCTAc 0–2	92.3	92.3	54.6	99.2	184.6	0.787
mCTAc 0–1	38.5	99.2	83.3	94.2	137.7	0.648
sCTAc 0–2	48	82.2	34.3	89.1	130.2	0.796
sCTAc 0–1	61.5	95.4	57.1	96.1	156.9	0.426

mCTAc, multiphase CT angiography collaterals; sCTAc, single-phase CT angiography collaterals; ASPECTS, Alberta stroke program early CT score; PPV, positive predictive value, NPV, negative predictive value; Y-index, Youden’s index.

**Figure 2:**
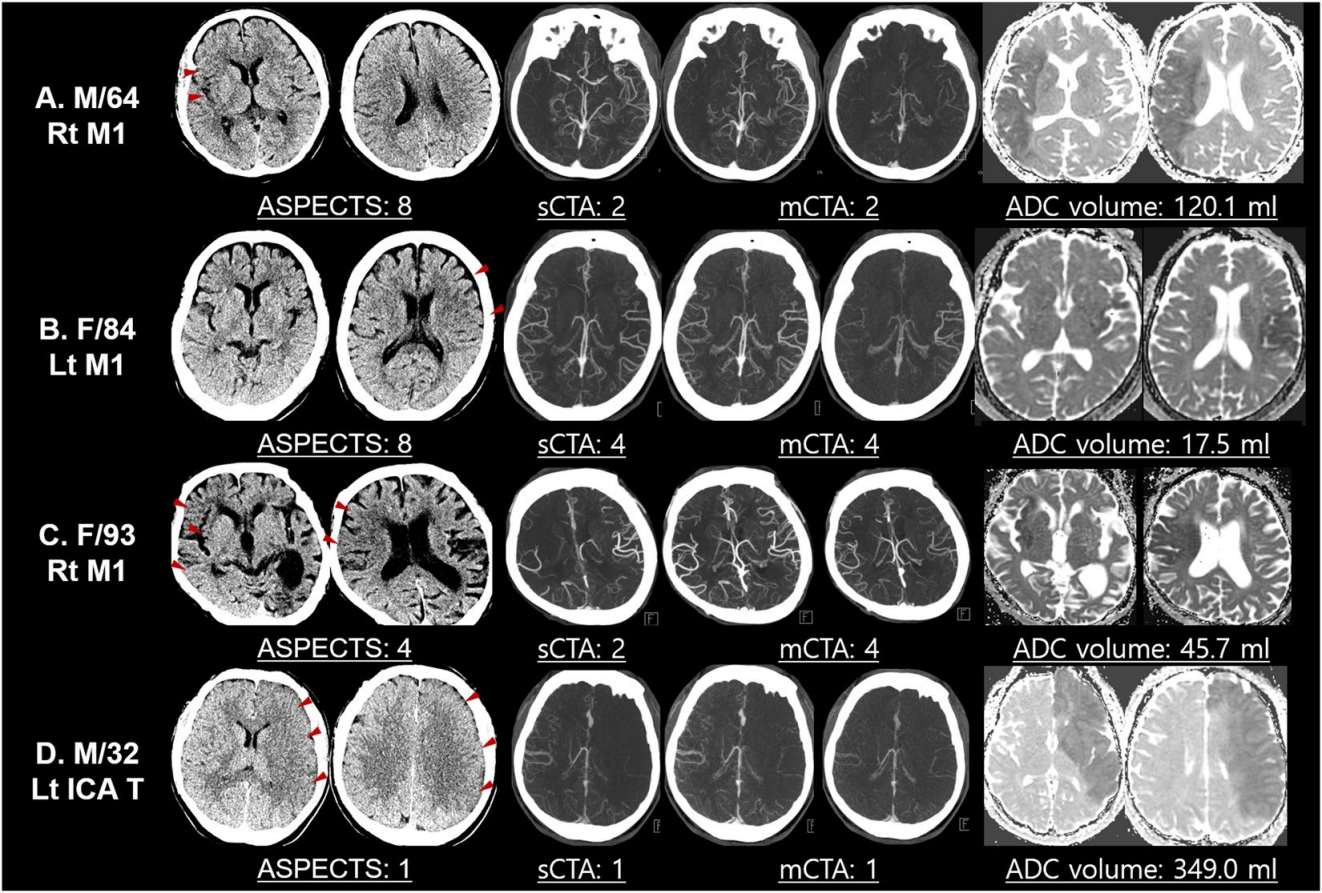
Illustrative cases of combined interpretation of ASPECTS and CTA-based collaterals in the estimation of infarct core volume. (**A**) While the ASPECTS score was high, it showed discrepancies with collateral grades. In this patient, MR ADC revealed a large infarct core. (**B**) This patient showed both a high ASPECTS score and CT collateral grade. MR ADC revealed a small infarct core, illustrating the importance of combined interpretation of ASPECTS and collateral grade. (**C**) While both ASPECTS and sCTAc grades suggested large infarct cores, mCTAc grade showed favorable pial collaterals. MR ADC revealed an intermediate infarct core. This case illustrated the importance of mCTAc for detecting larger infarct cores. (**D**) Low ASPECTS score and low collateral score predicted a high infarct core volume, as illustrated. ASPECTS, Alberta stroke program early CT score; CTA, computed tomographic angiography; MR, magnetic resonance; ADC, apparent diffusion coefficient; sCTAc, single-phase CT angiography collateral; mCTAc, multiphase CT angiography collateral.

For intermediate and larger infarct volume thresholds, a combined interpretation of collateral scores and ASPECTS score did not give significant added benefit for the prediction of infarct volume thresholds of <70 ml or ≥100 ml, but collateral imaging increased IRR for larger infarct thresholds. For the prediction of core volumes ≥100 ml, mCTAc 0–2 performed as well as ASPECTS 0–5 (Youden’s index; 184.6 vs. 174.5) with better inter-rater reliability (0.787 vs. 0.440). Examples of infarct core predictions using parameters obtained using mCTA are illustrated in Fig. 2.

## Discussion

The results of the present study indicate that by utilizing mCTA as a single modality, predictive capability can be improved by combining ASPECTS and collateral scores for smaller infarct volumes. For intermediate and larger infarct volumes, combining ASPECTS and collateral scores does not give added predictive ability. However, in larger infarct thresholds, mCTAc improves the agreement between raters compared to ASPECTS scores.

For the detection of smaller infarct cores, a combination of collateral grades and ASPECTS was significantly superior to ASPECTS alone. This was consistent with the current notion that, for later time windows, advanced imaging to selectively identify patients who would benefit from therapy is needed. A recent study agreed with our conclusion. In this study, a cerebral blood flow core volume of 50 ml was evaluated, in which NECT-ASPECTS ≥9 with a CTA collateral score of 3 had 100% specificity for identifying patients with a cerebral blood flow core volume ≤50 ml^14^. Our data further showed that the combined interpretation of ASPECTS and collateral scores in agreement is superior to using higher cutoff values of ASPECTS or collateral scores in terms of both predictive capability and IRR. This will be useful to facilitate patient selection for EVT utilizing mCTA.

To exclude larger infarct cores, combining ASPECTS and collateral scores did not give added predictive benefits. Contrary to our results, a recent study identified patients with infarct volumes >100 ml with high accuracy using both NECT ASPECTS 0–4 and a malignant CT collateral profile of absent collaterals in >50% of the M2 territory^15^. However, those authors used a DWI-based infarct volume. DWI volumes usually overestimate the infarct volume up to two-fold compared to ADC based core measurements^16^. Thus, the criteria of core volumes ≥100 ml based on ADC used in our study identify patients with larger cores than this study. Such difference may have been the reason for the inconsistent results.

While it failed to yield added predictive ability, an mCTA-based protocol could improve the agreement between readers in the intermediate and higher volume thresholds compared to ASPECTS alone. Low IRR^17^ and its nonlinear characteristics^18^ have indeed been reported as major limitations of ASPECTS. Reperfusion protocols for acute stroke patients are multidisciplinary and require close communication and agreement between neurology residents, stroke neurologists, and neurointerventionists to reduce in-hospital times and achieve better outcomes^19^. Improving IRR is at least as important as improving the predictive ability in this regard, and our results show that mCTA-based protocols can provide this added benefit.

Original reports on mCTAc show reduction of uncertainty in decision-making and a slightly better prediction of clinical outcomes^10^. The superiority of mCTAc over sCTAc could not be proven in our study, but when the results of predictive performance using cutoff values of mCTAc 0–2 and sCTAc 0–2 are compared, there is a tendency for mCTAc to perform better than sCTAc. This is reasonable, for it is generally thought that sCTAc may mislabel collateral scores due to its lack of temporal resolution^20^. This lack of temporal resolution will result in more stringent gradings for sCTAc, and more generous gradings for mCTAc, resulting in higher power for mCTAc in the larger infarct core ranges. Given the short image acquisition time and various further information the mCTA gives such as improved identification of occlusion sites^21^ or thrombus characteristics^22^, the authors advocate for the utilization of mCTA rather than conventional CT angiography.

This study had some limitations. The first was related to the inevitable time gap between CT and MRI, and areas of penumbra may evolve into ischemic cores during this interval. Therefore, CT perfusion-based core measurements are utilized. However, CT perfusion-based core measurements are reported to often overestimate^23^ and underestimate^24^ infarct volumes, and may also vary based on the method of analysis. Analysis of infarct volumes using ADC thresholds is still the gold standard^13^, and our results were significant in this regard. The second limitation was related to the retrospective design of the study and the limited number of patients. The study population included a small number of patients with large infarct volumes, most likely because large cores were excluded from the concomitant CT-MRI imaging pathway. Accordingly, the additive power of collateral imaging in large volume infarcts may have been reduced. Third, the time delay due to MRI can be an issue. Our institution uses a stepwise protocol of multiphase CT angiography for patient selection, and stroke MRI for further guidance of EVT before entering the angio suite. Total acquisition time is ideally 9 min 35 s. MRI can aid the procedure in various situations, such as identification of intracranial atherosclerosis-related occlusions^25^, but the time between imaging and reperfusion has a major impact on the patient, and we attempt to reduce this time at our institution.

In conclusion, mCTA can be utilized to improve the selection of patients with small cores beyond the usual selection criteria. We propose that a combination of ASPECTS 6–10 and sCTAc 3–5 may be used to predict infarct cores ≤31 ml. While it does not add predictive power in the larger core threshold, improved IRR can be achieved by using and mCTA-based protocol for the exclusion of patients with larger infarct cores.

## Supplementary information

Supplementary methods and supplementary tables

## Data Availability

The datasets generated during and/or analysed during the current study are available from the corresponding author on reasonable request.
