# Supplementary material for: Optimal Multiphase Computed Tomographic Angiography-based Infarct Core Estimations for Acute Ischemic Stroke
Source: Sci Rep. 2019 Oct 23;9:15243. doi: 10.1038/s41598-019-51708-6 (PMC6811584; doi:10.1038/s41598-019-51708-6)
Supplement: Supplementary file 1 — Supplementary methods and supplementary tables [file 41598_2019_51708_MOESM1_ESM.pdf]

Optimal Multiphase Computed Tomographic Angiography-based Infarct Core Estimations for Acute Ischemic Stroke

Seong-Joon Lee[1], Woo Sang Jung[2], Mun Hee Choi[1], Ji Man Hong[1], Jin Soo Lee[1], Jin Wook Choi[2*]

[1] Department of Neurology, Ajou University School of Medicine, Ajou University Medical Center, Suwon, South Korea

[2] Department of Radiology, Ajou University School of Medicine, Ajou University Medical Center, Suwon, South Korea

Short title: Multiphase CT Based Core Estimation

Corresponding author

Jin Wook Choi, MD, PhD

Department of Radiology, Ajou University School of Medicine

164, World cup-ro, Yeongtong-gu, Suwon-si, Gyeonggi-do, 16499, Republic of Korea

Tel.: +82-31-219-5834

Fax: +82-31-219-5862

E-mail: radjwchoi@gmail.com

Key words: endovascular treatment, acute ischemic stroke, multiphase computed tomography, infarct core

SUPPLEMENTARY MATERIALS

Supplementary methods

Imaging protocols

The CT scans (SOMATOM Definition Edge [128-channel] Siemens, Erlangen, Germany) including noncontrast and postcontrast axial parenchymal images, were acquired with contiguous 5-mm thick axial sections (120kV, 270mAs). CTA images were acquired from the aortic arch to the vertex with the following parameters: 100 kV, 180 mAs, 0.5 seconds per rotation, 0.5 pitch, and a 0.75-mm section thickness. For multiphase CT angiography, aortic arch vertex CT angiography performed with a multidetector CT scanner made up the first phase. Image acquisition was timed to occur during the peak arterial phase in the healthy brain and was triggered by bolus monitoring. The remaining two phases are from the skull base to the vertex in the equilibrium/peak venous and late venous phases in the healthy brain. Images were acquired with a 0.625-mm section thickness. The first phase of CT angiography from the arch to the vertex was acquired in less than 7 seconds, with an average dose length product of 700–800 mGy·cm. The second phase was acquired after a delay of 4 seconds that allows for table repositioning to the skull base. Scanning duration for each additional phase was 3.4 seconds. Thus, the three phases were each 8 seconds apart. A total of 80 mL of contrast material was injected at a rate of 5 mL/sec and followed by a 50-mL normal saline chase at a rate of 6 mL/sec. The CT source images were postprocessed to create coronal, sagittal, and axial multiplanar reformats in maximum intensity projection (MIP) images (10-mm slab and 2-mm interval) and volume-rendered 3D images.

MRI was performed with a 3T MR scanner (Intera Achieva; Philips Healthcare, Best, The Netherlands) using a 16-channel neurovascular (NV) head coil. The MRI protocol for acute stroke included the following sequences: localizer, DWI, T2 turbo spin echo, fluid-attenuated inversion recovery, gradient echo, and perfusion-weighted imaging. Total

acquisition time is ideally 9minutes 35 seconds. The DWI sequences were single-shot echo planar imaging acquired in the axial plane with the following parameters: TR 3000 ms; TE 80 ms; b value 1000 s/mm^2 ; field of view 22 cm; matrix size 128   128; and 28 slices with 5-mm slicethickness and without interslice gap. Apparent diffusion coefficient (ADC) values were calculated at 2 different b values (b=0 and b= 1000 s/mm^2). DWI images and ADC maps were generated on the scanner console at the time of imaging.

Supplementary Table 1. Characteristics of the included acute ischemic stroke population.

**Patients**	N=142
Baseline characteristics	
Sex, male	81 (57.0%)
Age	70.0 ± 12.9
HTN	80 (56.3%)
DM	25 (17.6%)
Atrial fibrillation	63 (44.4%)
Baseline NIHSS	17.0 [13.75 – 19.25]
Occlusion location	
ICA T	35 (24.6%)
ICA I	12 (8.5%)
MCA M1	75 (52.8%)
MCA M2	20 (14.1%)
**Acute treatments**	
Onset to door, min	229 ± 232
Onset to CT, min	249 ± 239
CT to MRI, min	51.6 ± 12.3
IV tPA	62 (43.7%)
Endovascular treatment	115 (81.0%)
Outcomes	
Good Outcome (mRS 0~2)	75 (52.8%)
Futile Outcome (mRS 5~6)	28 (19.7%)

HTN, hypertension; DM, diabetes mellitus; NIHSS, National Institute of Health Stroke Scale; ICA, internal carotid artery; MCA, middle cerebral artery; CT, computed tomography; MRI, magnetic resonance imaging; IV tPA, intravenous tissue plasminogen activator; mRS, modified Rankin Scale.

Supplementary Table 2. Factors associated with good outcomes in the subgroup of patients that performed endovascular treatment.

	Good outcomes* (N=63)	Poor outcomes (N=52)	P
Age	68 ± 12	74 ± 12	0.016
Sex, male	35 (55.6%)	29 (55.8%)	0.982
HTN	37 (58.7%)	33 (63.5%)	0.605
DM	15 (23.8%)	7 (13.5%)	0.160
Atrial fibrillation	26 (41.3%)	28 (53.8%)	0.179
NIHSS, median	16.0 [12.0 – 18.0]	19.0 [16.0 – 20.75]	<0.001
ASPECTS, median	8.0 [6.0 – 9.0]	7.0 [5.0 – 8.0]	0.005
sCTAc, median	3.0 [3.0 – 4.0]	3.0 [20 – 4.0]	0.067
mCTAc, median	4.0 [3.0 – 4.0]	4.0 [3.0 – 4.0]	0.029
Core volume, ml	21 ± 25	46 ± 63	0.008
Core <31ml	50 (79.4%)	33 (63.5%)	0.058
Core < 70 ml	59 (93.7%))	41 (78.8%)	0.019
Core ≥ 100ml	2 (3.2%)	7 (13.5%)	0.041
Occlusion location			0.008
ICA I	1 (1.6%)	10 (19.2%)	
ICA T	17 (27.0%)	13 (25.0%)	
MCA M1	39 (61.9%)	22 (42.3%)	
MCA M2	6 (9.5%)	7 (13.5%)	
Reperfusion			
IV thrombolysis	31 (49.2%)	18 (34.6%)	0.115
Onset to puncture, min	316 ± 189	338 ± 262	0.248
Reperfusion success	62 (98.4%)	43 (82.7%)	0.003
Hemorrhagic transformation			0.115
None	42 (66.7%	26 (50.0%)	
HI 1	9 (14.3%)	5 (9.6%)	
HI 2	8 (12.7%)	10 (19.2%)	
PH 1	2 (3.2%)	6 (11.5%)	
PH 2	2 (3.2%)	5 (9.6%)	

*Good outcomes are classified as 3 month modified Rankin Scale of 0 ~ 2.

HTN, hypertension;DM, diabetes mellitus; NIHSS, National Institute of Health Stroke Scale; ASPECTS, Alberta Stroke Program Early CT score; sCTAc, single phase computed

tomographic angiography collateral score; mCTAc, multiphase computed tomographic angiography collateral score; ICA, internal carotid artery; MCA, middle cerebral artery; IV, intravenous, HI, hemorrhagic infarct; PH, parenchymal hematoma.

Supplementary Table 3. Multivariate analysis models validating the clinical significance of various parameters in prediction of good functional outcomes (3 month modified Rankin Scale of 0 ~2) in the subgroup of patients that performed endovascular treatment.

**Model 1**	**OR (95% CI)**	**P**
ADC core volume	0.98 [0.97 – 0.99]	0.038
Age	0.94 [0.89 – 0.99]	0.764
NIHSS	0.76 [0.65 – 0.89]	0.001
Occlusion location		0.042
MCA M1	Reference	
ICA I	0.02 [0.00 -0.34]	0.008
ICA T	1.39 [0.43 – 4.52]	0.584
MCA M2	0.50 [0.09 – 2.65]	0.414
Successful reperfusion	85.56 [2.56 – 2858.54]	0.013
PH2	0.48 [0.03 – 6.58]	0.579
**Model 2**		
Core <31 ml	2.14 [0.65 – 7.03]	0.208
Age	0.94 [0.86 – 0.99]	0.011
NIHSS	0.75 [0.64 – 0.87]	<0.001
Occlusion location		0.050
MCA M1	Reference	
ICA I	0.01 [0.00 – 0.32]	0.008
ICA T	1.09 [0.36 – 3.27]	0.883
MCA M2	0.45 [0.09 – 2.33]	0.339
Successful reperfusion	83.58 [2.44 – 2859.80]	0.014
PH2	0.18 [0.02 – 1.51]	0.115
**Model 3**		
Core <70 ml	4.76 [0.91 – 24.85]	0.064
Age	0.94 [0.90 – 0.99]	0.014
NIHSS	0.74 [0.63 – 0.86]	<0.001
Occlusion location		0.046
MCA M1	Reference	
ICA I	0.01 [0.00 – 0.33]	0.014
ICA T	1.40 [0.43 – 4.55]	0.575
MCA M2	0.53 [0.10 – 2.78]	0.448
Successful reperfusion	78.30 [2.31 – 2650.96]	0.015
PH2	0.209 [0.02 – 2.05]	0.209
**Model 4**		
Core ≥100 ml	0.28 [0.03 – 2.69]	0.269
Age	0.94 [0.90 – 0.99]	0.015
NIHSS	0.75 [0.64 – 0.87]	<0.001
Occlusion location		0.049
MCA M1	Reference	
ICA I	0.02 [0.00 – 0.34]	0.008
ICA T	1.14 [0.37 – 3.51]	0.820
MCA M2	0.45 [0.09 – 2.26]	0.330
Successful reperfusion	66.55 [2.08 – 2128.48]	0.018

PH2	0.22 [0.03 – 2.69]	0.269

ADC, apparent diffusion coefficient; NIHSS, National Institute of Health Stroke Scale; ICA, internal carotid artery; MCA, middle cerebral artery; PH, parenchymal hematoma.
